# Analysis of Lung Metastases in Patients with Primary Extremity Sarcoma

**DOI:** 10.1080/13577140310001607284

**Published:** 2003-06

**Authors:** N. Songür, M. Dinç, C. Özdilekcan, S. Eke, U. Ok, M. Oz

**Affiliations:** 1 Department of Chest Diseases Cancer Research Hospital Ankara 06660 Turkey; 2 Department of Orthopedics Cancer Research Hospital Ankara Turkey; 3 National Institute on Drug Abuse Cellular Neurobiology Section Baltimore MD USA

## Abstract

*Purpose*: To investigate the incidence, radiographic findings, and the time course for the appearence of lung metastases from primary extremity sarcoma.

*Patients and methods*: Four-hundred patients with extremity sarcoma were evaluated retrospectively for lung metastases. Multiple clinical factors were analyzed for possible influence on the metastases-free interval and subsequent metastases development. Radiographic findings of metastases were also reviewed.

*Results*: Ninety of 400 patients (23%) developed lung metastases. Median time from presentation to detection of metastasis was 8.0 ± 1.1 months (95% CI: 6.02–9.98). More than 75% of patients developed metastases within 1 year after presentation of the primary sarcoma. As disease grade increased, the metastases-free intervals shortened significantly. Histologically, synoviosarcoma and osteogenic sarcoma were more often associated with the development of lung metastases. In this subgroup, 54 patients (60%), the presence of solitary (11) or multiple (43) nodular metastases was the only radiological finding. In 36 of 90 patients (40%), parenchymal mass, pleural effusion, hilar lymphadenopathy and pneumothorax were found.

*Conclusions*: Twenty-three percent of patients in our study developed lung metastases. As the grade of the disease increased, metastases-free intervals are shortened. Although it has been reported that lung metastases in patients with extremity sarcomas may present as solitary or multiple nodules in earlier trials with radiographic screening methods, the current review of 400 patients found that a substantial number of patients may present radiological appearances other than nodular formations.


*Sarcoma*, June 2003, *VOL. 7, NO. 2, 63–67*

Taylor & Francis healthsciences

ORIGINAL ARTICLE

### Analysis of lung metastases in patients with primary extremity sarcoma

N. SONGÜR[1], M. DINÇ[1], C. ÖZDILEKCAN[1], S. EKE[2], U. OK[1] & M. OZ[3]

*[1]Department of Chest Diseases, Cancer Research Hospital, Ankara, Turkey, [2]Department of Orthopedics, Cancer Research Hospital, Ankara, Turkey and [3]National Institute on Drug Abuse, Cellular Neurobiology Section, Baltimore, MD, USA*

**Abstract**

*Purpose*: To investigate the incidence, radiographic findings, and the time course for the appearence of lung metastases from primary extremity sarcoma.

*Patients and methods*: Four-hundred patients with extremity sarcoma were evaluated retrospectively for lung metastases. Multiple clinical factors were analyzed for possible influence on the metastases-free interval and subsequent metastases development. Radiographic findings of metastases were also reviewed.

*Results*: Ninety of 400 patients (23%) developed lung metastases. Median time from presentation to detection of metastasis was $8.0 \pm 1.1$ months (95% CI: 6.02–9.98). More than 75% of patients developed metastases within 1 year after presentation of the primary sarcoma. As disease grade increased, the metastases-free intervals shortened significantly. Histologically, synoviosarcoma and osteogenic sarcoma were more often associated with the development of lung metastases. In this subgroup, 54 patients (60%), the presence of solitary (11) or multiple (43) nodular metastases was the only radiological finding. In 36 of 90 patients (40%), parenchymal mass, pleural effusion, hilar lymphadenopathy and pneumothorax were found.

*Conclusions*: Twenty-three percent of patients in our study developed lung metastases. As the grade of the disease increased, metastases-free intervals are shortened. Although it has been reported that lung metastases in patients with extremity sarcomas may present as solitary or multiple nodules in earlier trials with radiographic screening methods, the current review of 400 patients found that a substantial number of patients may present radiological appearances other than nodular formations.

**Key words:** *primary extremity sarcoma, lung metastases*

### Introduction

Primary extremity sarcomas are unique in that, in the majority of cases, early metastases are confined to the lungs. Patients with extremity sarcomas are more likely to have distant metastatic disease as their initial site of recurrence, whereas those with retroperitoneal and visceral sarcomas tend to have local recurrence.[1] Consequently, patients with primary soft-tissue sarcoma (STS) in an extremity tend to develop pulmonary metastases more frequently than those patients with sarcomas located in other sites.[2,3] Approximately 20% of patients with extremity STS will develop isolated pulmonary metastases during the course of their disease.[4]

Although considerable progress has been made in both chemotherapy and surgical treatment of extremity sarcoma, pulmonary metastases in these patients remain a major cause of mortality. Since the diagnosis of pulmonary metastases significantly effects the outcome of treatment and prognosis, detecting such pulmonary metastases is vitally important.[5] Currently, the characteristics of the histological and radiographic findings, the relationship between these findings, and the subsequent clinical course of the pulmonary metastases remains largely unknown.

In the present study, we have analysed retrospective data relevant to pulmonary metastases in a large series of adult patients with extremity sarcomas. We have attempted to evaluate the true incidence, the time of occurrence, and radiographic appearance of pulmonary metastases from extremity sarcomas.

### Patients and methods

For this study, we included all adult patients (age >16 years old) with a primary extremity sarcoma who were admitted and treated at the Cancer Research Hospital (Ankara, Turkey) from

Correspondence to: Necla Songür, M.D., Kennedy cad. No. 24/11, Kavaklidere, Ankara 06660, Turkey. Tel.: +90-312-466-4383; Fax: +90-312-466-4041; E-mail: yildiransongur@hotmail.com

ISSN 1357-714X print/1369–1643 © 2003 Taylor & Francis Ltd
DOI: 10.1080/13577140310001607284

March 1990 through November 2000. During this interval, 400 patients with primary extremity sarcomas were evaluated. Of these, 90 patients developed lung metastases. These patients comprise the study group for this report. We reviewed the chest radiographs from the initial diagnosis at the time of the primary tumour and chest radiographs and computed tomography (CT) performed at time of metastases. All radiographs were reviewed independently by at least two of the authors.

Patients with extremity sarcoma were followed up by routine postoperative chest X-rays and CT scanning obtained every 4 months for the first 2 years. During the third through fifth follow-up years, patients received chest X-ray and CT scans every 6 months. After 5 years postoperatively, patients were followed-up by yearly CT scans. All CT scans (General Electric Sytec 4000i, Milwaukee, USA) were obtained with abutting 5- or 10-mm sections through the lungs. The radiographic findings of lung metastases were evaluated. The data were obtained from the first positive chest radiographs and CT. Morphological characterization of the pulmonary metastases included a measurement of the diameter of the largest mass, the determination of the total number of lesions and the description of their appearance. In this study, pulmonary nodules were considered to be $< 3$ cm in diameter. Parenchymal mass was considered to be $> 3$ cm in diameter, with irregular contour. We also looked for a smooth or spiculated contour, the presence or absence of cavitation and other associated features such as calcification, hilar and mediastinal adenopathy or pleural effusion. If a solitary nodule or mass was present on the first abnormal radiography, confirmation of metastatic disease was obtained by biopsy and/or surgical exploration. In cases in which clinical evidence for a second primary tumour could not be found, the presence of more than one parencyhmal nodule was considered metastatic, and originating from the extremity sarcoma.

The histopathological findings, including subtypes and grades, were reviewed and diagnoses were confirmed by an experienced author. We assessed the metastases-free interval. Multiple clinical factors were analysed for possible influence on the metastases-free interval and subsequent metastases development. Factors included patient age, gender, the grading at initial presentation, histopathology, the location (upper or lower extremity) and treatment (preoperative chemotherapy, preoperative radiotherapy, postoperative chemotherapy, postoperative radiotherapy). Patients who had $\leq 1$ year between diagnosis of the primary tumor and diagnosis of lung metastases were compared with patients who had a metastases-free interval of $>1$ year. One year was chosen, since more than 75% of patients developed lung metastases within 1 year after presentation of the primary sarcoma.

*Statistical analysis*

Statistical analyses and descriptive tables were made by computer software (SPSS version 7.5). The comparison of sarcoma subgroups was made using chi-square tests. The metastases-free intervals for the groups were calculated with Kaplan–Meier survival analyses.[6] The comparison of the survival rates of all three grades were calculated with the log-rank test. Stepwise logistic regression analysis was performed to determine the influence of specific factors on the metastases-free interval. In all analyses, $P < 0.05$ was considered statistically significant.

### Results

The median age was 43 years (range 16–79 years). The median length of follow-up after diagnosis of the primary extremity sarcoma was 26.1 months. The median time elapsed from presentation of extremity sarcoma to detection of subsequent lung metastases was $8.0 \pm 1.01$ months (95% CI: 6.02–9.98). Within 1 year following presentation, more than 75% of the patients developed lung metastases.

We have analysed the relationship between the grade of primary tumours and the median time elapsed from presentation to detection of lung metastases. The median time for developing pulmonary metastases was $14 \pm 7.3$ (95% CI: 0–28.4), $11 \pm 0.78$ (95% CI: 9.48–12.52) and $4 \pm 0.88$ (95% CI: 2.28–5.72) months in patients with grade I, II and III, respectively. The summary of the results on the grade of the primary tumours is presented in Table 1. The metastases-free intervals lessened significantly as the disease grade increased ($P < 0.001$). Results indicating the time elapsed from presentation to detection of lung metastases for each grade are presented in Fig. 1. The results of multivariate logistic regression analysis investigating influence of clinical factors on metastases-free interval are shown in Table 2. Results indicated that only tumour grade was significant ($P = 0.003$).

Histopathological examination of the metastatic specimens found a wide spectrum of different histological types (Table 3). Analysis of the distribution of primary histology demonstrated that the most common type of extremity sarcoma was malignant fibrous histiocytoma (MFH; 33.7%); followed by liposarcoma (23.7%) and osteogenic

**Table 1.** *Grade at presentation of extremity sarcomas in the patients who developed pulmonary metastases*

| Grade | *n* |
|---|---|
| I | 10 |
| II | 44 |
| III | 36 |
| Total | 90 |

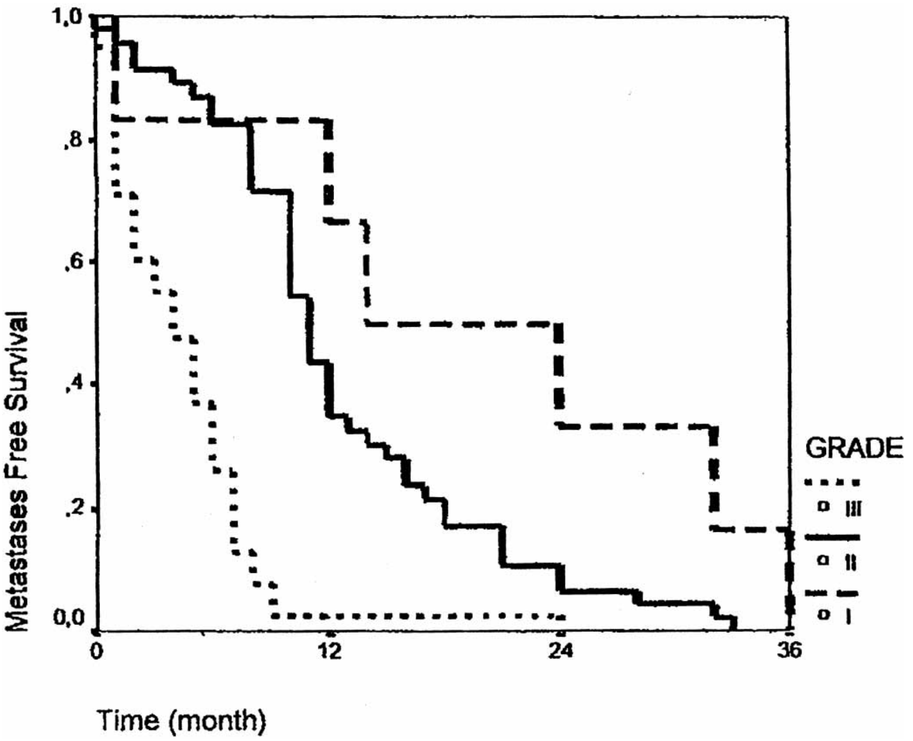

**Fig. 1.** *The time course of the metastases-free intervals for each grade of disease.*

**Table 2.** *Multivariate logistic regression analysis of clinical factors on metastases-free interval*

| Variable | $\beta$ | SE | Wald | *P* value | OR | %95 CI |
|---|---|---|---|---|---|---|
| Grade (II) | −2.2714 | 0.9707 | 5.4755 | 0.0193 | 0.1031 | 0.0154–0.6915 |
| Grade (III) | −2.8759 | 0.9588 | 8.9973 | 0.0027 | 0.0564 | 0.0086–0.3691 |

SE, standard error; OR, odds ratio; CI, confidence interval.

**Table 3.** *Summary of the results on histological types and the number of pulmonary metastases in 400 adult patients with extremity sarcoma*

| Histological type | Overall *n* (%) | With pulmonary Metastases *n* (%) | *P* value |
|---|---|---|---|
| Malignant fibrous histiocytoma | 135 (33.7) | 20 (22.2) | 0.022* |
| (Myxoid and pleiomorph) liposarcoma | 95 (23.7) | 14 (15.5) | 0.077 |
| Fibrosarcoma | 16 (4) | 6 (6.6) | 0.32 |
| Synoviosarcoma | 13 (3.25) | 6 (6.6) | 0.16 |
| Leiomyosarcoma | 22 (5.5) | 7 (7.7) | 0.65 |
| Rhabdomyosarcoma | 26 (6.5) | 9 (10) | 0.42 |
| Malignant schwannoma | 19 (4.75) | 7 (7.7) | 0.3 |
| Extraskeletal osteosarcoma | 5 (1.25) | 2 (2.2) | 0.62 |
| Extraskeletal chondrosarcoma | 4 (1) | 1 (1.1) | 0.4 |
| Hemangiopericytoma | 3 (0.75) | 1 (1.1) | 0.66 |
| Osteogenic Sarcoma | 42 (10.5) | 17 (18.8) | 0.066 |
| Total | 400 | 90 | |

*Indicates statistically significant difference at the level of <0.05.

sarcoma (10.5%). Synoviosarcoma and osteogenic sarcoma were more often associated with the development of pulmonary metastases than other histological types (46 and 40.4%, respectively).

*Radiological appearances*

Evaluations of radiological appearence were based on the chest radiographs and CTs of 90 patients with extremity sarcoma. CT examinations indicated that in 54 of 90 patients (60%), metastases were confined to the presence of solitary (11/90) or multiple (43/90) nodular metastases. In 11 patients with solitary metastases, all pulmonary nodules were >5 mm in diameter. In 43 patients with multiple nodular metastases, the size of the nodules showed significant variations. Of this subgroup, 32 patients had nodules both greater and less than 5 mm in diameter. In seven patients, all metastases were <5 mm in diameter. In the remaining four patients, all metastases were >5 mm in diameter. Forty percent of the metastases presented in different radiological appearances other than nodular formations. The summary of the results on the radiological manifestation of the metastatic findings is presented in Table 4.

The distributions of the metastatic nodules in chest radiographs and CTs were not uniform. The metastases were most commonly located in the peripheral and lower zones of the lung. All of the patients with solitary metastases had STS ($n = 11$). In seven of 11 patients, diagnosis was confirmed by thoracotomy. The physical conditions of the remaining four patients prevented surgical exploration, and follow-up CTs indicated disease progression despite the chemotheraphy. As the radiographic appearance of lung metastases, patients with osteogenic sarcoma had parenchymatous or subpleural nodules, which were often ossified. All masses had smooth borders with no cavitation. In 14 patients, pleural effusions were also noted. Parenchymal lesions were based on pleura. Pleural effusions were found as isolated radiographic findings in two of the patients. The diagnosis of metastases was not confirmed by pleural biopsy and/or surgical exploration in one of patients since the physical condition of the patient was not suitable for these procedures. However, CTs of this patient indicated a marked increase in the size of the pleural effusions. Histological and/or cytological confirmation of the metastatic disease was obtained by biopsy in another patient. In one patient, pneumothorax (without any radiograpic indication of nodule) was found in the first presentation. Hilar lymphadenopathy was the only feature of metastasis in one patient. In this case, diagnosis was made by mediastinoscopy.

### Discussion

In our study, 90 out of 400 patients (23%) with extremity sarcoma developed pulmonary metastases during a 10-year follow-up period. The predilection for pulmonary metastases in patients with extremity sarcoma has also been shown in other studies.[3,7–9] In an earlier study reviewing 211 patients with high-grade extremity sarcoma, a recurrence rate of 31%, including both local and distant metastases, was reported.[3] In that study, pulmonary metastases alone represented 70% of these sites, with an incidence of 21% in those patients with high-grade extremity STS. In another investigation, 135 of 716 (19%) patients of extremity sarcoma were found to have pulmonary metastases.[4] The findings of our study are in close agreement with the results of earlier reports.[3,4]

The time from presentation to development of pulmonary metastases is difficult to ascertain from the literature. In our study, more than 75% of patients developed lung metastases within 1 year after presentation.

The probability of the occurrence of pulmonary metastases was significantly influenced by the histopathological type of the tumour.[5,10,11] In a study of 91 patients with extremity STS, those with MFH, malignant schwannoma and spindle cell sarcoma had an increased incidence of pulmonary metastases.[8] In our study, histological analysis of primary lesions indicates that pulmonary metastases arise most commonly in patients with synoviosarcoma and osteosarcoma. Our study also suggested a relationship between metastases-free intervals and disease grade. A longer metastases-free interval is seen in lower grade tumours. Since the majority of patients developed lung metastases within 1 year, the optimal follow-up for lung imaging should be chest X-rays and CT scanns every 4 months during the first 2 years after diagnosis.

**Table 4.** *Manifestation of metastatic lung disease in 90 patients as revealed on computed tomography*

| Manifestation | *n* | % |
|---|---|---|
| Solitary nodule | 11 | 12.2 |
| Bilateral multiple nodules | 43 | 47.7 |
| Pulmonary parenchymal mass | 10 | 11.1 |
| Pleural effusion + pulmonary parenchymal mass | 8 | 8.8 |
| Pleural effusion | 2 | 2.2 |
| Pulmonary parenchymal mass + multiple nodules | 10 | 11.1 |
| Pulmonary parechymal mass + multiple nodules + pleural effusion | 4 | 4.4 |
| Hilar lymphadenopathy | 1 | 2.2 |
| Pneumothorax | 1 | 1.1 |
| Total | 90 | 100% |

There are only a few anectodal reports in the literature describing the radiographic appearances of the pulmonary metastases from extremity sarcoma. In general, these studies suggest that the pulmonary metastases present as solitary or multiple pulmonary nodules.[12–14] In our study, the most frequent (47.7%) manifestation of metastasis on CT was multiple pulmonary nodules. This finding is comparable to the results reported in earlier studies.[12–15] The pulmonary nodules were concentrated mainly in the lower lobes of the lung as subpleural or parenchymatous. In patients with STS, most pulmonary nodules were subpleural, whereas patients with osteogenic sarcoma had nodules that were paranchymatous or subpleural and often ossified. A sharp and well-circumscribed margin was usually seen in these nodules. Considering that sarcoma patients have higher risk of metastases, in cases where clinical proof for a second primary tumour was missing or ambiguous, the presence of more than one parenchymal nodule was considered metastatic. This pattern of pulmonary metastasis is not unique to patients with extremity sarcoma. Metastases from hypernephromas, testicular, breast, cervical and endometrial carcinomas may all have a similar pattern. In the current study, it was noted that all of the solitary metastases were detected in the patients with extremity STS. Our study demonstrates that patients with extremity sarcomas have also different radiological appearances, such as pulmonary mass, pleural effusion, hilar lymphadenopathy and pneumothorax. To our knowledge, this is one of the largest studies focusing on all radiological features of the pulmonary metastases of extremity sarcomas.

Another notable finding concerns the state of the metastases. At the time of the initial assesment, all lesions are metastatic and active, but they may disappear subsequently or become totally necrotic with chemotheraphy.[16] Although complete necrosis or disappearance of the metastatic nodule(s) is possible, this did not occur in our study. Since our study was conducted in a retrospective manner, pathological confirmation of suspicious lesions, especially multiple nodules on chest CTs or cytological proof of pleural effusions were not always present. However, follow-up of all lesions seen on CTs was performed.

In conclusion, in our study, we found an incidence of lung metastases of approximately 23% in patients with extremity sarcomas. Most patients developed lung metastases within 1 year after presentation. Metastases-free intervals were significantly shortened as the grade of the disease increased. Radiographically, pulmonary metastases in patients with extremity sarcomas may present as solitary or multiple nodules most commonly located in the peripheral and lower zones of the lung. However, it should also be noted that a substantial number of patients may present with radiological appearances of lesions that are neither solitary nor nodular.

### Acknowledgements

The authors thank John Schmittner, M.D., and Mrs. Marry Pfeiffer of N.I.H., N.I.D.A./I.R.P., for their valuable suggestions on the manuscript.

### References

1. Brennan MF. Management of extremity soft tissue sarcoma. *Am J Surg* 1989; 158: 71–8.
2. Vezeridis MP, Moore R, Karakousis CP. Metastatic patterns in soft tissue sarcomas. *Arch Surg* 1983; 118: 915–8.
3. Potter DA, Glenn J, Kinsella T, Glatstein E, Lack EE, Restepo C, *et al.* Patterns of recurrence in patients with high-grade soft tissue sarcomas. *J Clin Oncol* 1985; 3: 353–66.
4. Gadd M, Casper E, Woodrof JM, McCormack PM, Brennan MF. Development and treatment of pulmonary metastases in adult patients with extremity soft tissue sarcoma. *Ann Surg* 1993; 218: 705–12.
5. Putman JB, Roth JA, Wesley MN, Johnston MR, Rosenberg SA, *et al.* Survival following aggressive resection of pulmonary metastases from osteogenic sarcoma: analysis of prognostic factors. *Ann Thorac Surg* 1983; 36: 516–23.
6. Kaplan E, Meier P. Nonparametric estimation from incomplete observations. *J Am Stat Assoc* 1958; 53: 457–62.
7. Huang MN, Edgerton F, Takita H, Douglas HO Jr, Karakousis C. Lung resection for metastatic sarcoma. *Am J Surg* 1978; 135: 804–6.
8. Markhede G, Angervall L, Steiner B. A multivariate analysis of the prognosis after surgical treatment of malignant soft tissue tumours. *Cancer* 1982; 49: 1721–33.
9. Cantin C, McNeer GP, Chu FC, Booher RJ. The problem of local recurrence after treatment of soft tissue sarcoma. *Ann Surg* 1968; 168: 47–53.
10. Chang HR, Gaynor J, Tan C, Hajdu SI, Brennan MF. Multifactorial analysis of survival in primary extremity liposarcoma. *World J Surg* 1990; 14: 610–8.
11. Brennan MF, Casper ES, Harrison LB, Shiu MH, Gaynor J, Hadju SI. The role of multimodality therapy in soft tissue sarcoma. *Ann Surg* 1991; 168: 47–53.
12. Pass HI, Dywer A, Makuch R, Roth JA. Detection of pulmonary metastases in patients with osteogenic and soft tissue sarcomas: The superiority of CT scans compared with conventional linear tomograms using dynamic analysis. *J Clin Oncol* 1985; 3: 1261–5.
13. Vanel D, Henry-Amar M, Lumbrosa J, *et al.* Pulmonary evaluation of patients with osteosarcoma: roles of standart radiography, CT, scintigraphy, and tomoscintigraphy. *Am J Radiol* 1984; 143: 519–23.
14. Martine P, Herman IL. Pulmonary metastatic disease: radiologic-surgical correlation. *Radiology* 1987; 164: 719–22.
15. Crow J, Slavin G, Kree L. Pulmonary metastases: a pathologic and radiologic study. *Cancer* 1981; 47: 25–95.
16. Hidalgo H, Korobkin M, Kinney TR, Falleta J, Heaston DH, Kirks DR. The problem of benign pulmonary nodules in children receiving cytotoxic chemotheraphy. *Am J Radiol* 1983; 140: 21–4.
